# Do Polish Konik Horses Find Soaked Hay to Be Palatable?

**DOI:** 10.3390/ani16111663

**Published:** 2026-05-29

**Authors:** Ewelina Tkaczyk, Izabela Ewelina Gazda, Michalina Humięcka, Jarosław Łuszczyński, Kinga Basińska, Beata Kaczmarek, Joanna Barłowska, Przemysław Tkaczyk, Iwona Janczarek

**Affiliations:** 1 Department of Horse Breeding and Use, University of Life Sciences in Lublin, Akademicka 13, 20-950 Lublin, Poland; ewelina.tkaczyk@up.edu.pl (E.T.); misia_humiecka@interia.pl (M.H.); iwona.janczarek@up.edu.pl (I.J.); 2 Department of Genetics, Animal Breeding and Ethology, Faculty of Animal Science, University of Agriculture in Cracow, 30-059 Cracow, Poland; jaroslaw.luszczynski@urk.edu.pl (J.Ł.); kingasack@gmail.com (K.B.); 3 Sub-Department of Internal Diseases of Farm Animals and Horses, Faculty of Veterinary Medicine, University of Life Sciences in Lublin, 20-612 Lublin, Poland; beata.kaczmarek@up.edu.pl; 4 Department Quality Assessment and Processing of Animal Products, University of Life Sciences in Lublin, Akademicka 13, 20-950 Lublin, Poland; joanna.barlowska@up.edu.pl; 5 Department of Agricultural and Environmental Chemistry, University of Life Sciences, Akademicka 13, 20-950 Lublin, Poland; przemyslaw.tkaczyk@up.edu.pl

**Keywords:** Polish Konik horse, hay, soaking in water, feeding behavior, palatability

## Abstract

Frequently, equine diseases necessitate the moistening or soaking of hay for the horses. It was assumed that this treatment would not reduce the willingness of the Polish Konik horses with low nutritional requirements to consume the hay. Furthermore, it was believed that moistening would increase the palatability of this feed. The aim of this study was to determine time and frequency parameters and the amount of unconsumed dry, moist, or soaked hay. The research material comprised 12 Polish Konik horses consuming either 1 kg of dry hay, 2.1 kg of moist hay, or 3.3 kg of soaked hay on three subsequent days of the experiment. The following parameters were determined: the time of hay intake, the duration and frequency of pauses in intake, the time of straw intake, the time and frequency of drinking, and the time spent on other activities during a one-hour period following the provision of hay, after which the feed refusals were weighed. An algorithm for assessing the ‘palatability’ of hay was also developed. The experimental time of hay intake ranged from 1261.67 s (moist hay) to 2663.33 s (soaked hay), and the maximum mass of feed refusals ranged from 10.00 g (dry hay) to 343.33 g (soaked hay). No sex-related differences were noted. The time of straw intake did not exceed 60 s. In general, Polish Konik horses were noted to be selective in their feeding habits, as the ‘palatability’ of soaked hay was low at best, that of dry hay was moderate or high, and that of moist hay was very high. The low ‘palatability’ of soaked hay was more evident in the geldings than in the mares. The reduced willingness to consume hay did not contribute to an increase in the straw intake, which is not a substitute for roughage. It is also important to note that different results may be expected in some horses, as the values for the traits examined indicated significant individual variation.

## Simple Summary

Primarily for health reasons, it is sometimes necessary to moisten or soak horse hay in water. This study hypothesized that if such hay is provided to Polish Konik horses, which are a primitive horse breed with low nutritional requirements, their willingness to consume it would not decrease. It was found, however, that hay soaked in water was not palatable to the horses, particularly the geldings, who consumed it for a shorter duration and in smaller quantities than the mares. On the contrary, hay moistened with water was consumed at least as readily as dry hay. Overall, the ‘palatability’ of soaked hay was rated as low at best, which may indicate that Polish Konik horses are, after all, at least partially selective. Dry hay was, generally, moderately or highly palatable, while moist hay was very highly palatable. However, the aversion to soaked hay did not lead to increased intake of bedding straw.

## 1. Introduction

Horses often face challenges associated with domestication [1,2,3]. Occasionally, welfare issues arise, such as those resulting from restrictions on the five freedoms [2,4,5]. Conversely, in many cases, animals receive excessive care, which can diminish their natural ability to cope in environments created by humans. This leads to deterioration in the animals’ health, for example, resulting in the development of so-called lifestyle diseases, including respiratory and metabolic disorders [2,6,7].

Equine dietary requirements clearly include the necessity of roughage [1,3,5,8,9,10]. This is indicated primarily by the structure of their digestive system but also by their natural adaptation to feeding for most of the day. Unfortunately, the caretakers of these animals are increasingly seeking more specific nutritional solutions to replace traditional hay, which is often considered a factor contributing to many health problems in the animals in their care [11,12,13]. On the contrary, horses should be provided with hay of the highest possible quality, precisely to prevent health problems [14,15]. For example, contaminated hay can contribute to sand accumulation, i.e., the build-up of significant amounts of sand and soil in the digestive tract [16,17], leading to frequent colic episodes. Contaminated or dusty hay also negatively impacts the respiratory system [11]. In this case, soaking the hay will reduce the amount of dust and particles in the air, which is advisable for all horses kept in stables and absolutely essential for horses with asthma [18]. Symptoms of this condition include, among others, the accumulation of excess mucus in the airways, paroxysmal coughing, general malaise, and a decline in physical performance.

However, daily treatment of hay, whether through moistening, steaming, rinsing, or even soaking, is necessary not only when contamination occurs but also in many other situations. Nevertheless, it is worth noting that soaking hay, in particular, results in the leaching of nutrients, namely non-structural carbohydrates (NSCs), macro- and microminerals, and vitamins, which is, generally, an undesirable phenomenon [19,20]. This process, however, does not affect the protein content. The reduction in the levels of the above-mentioned components is dependent on the duration of soaking, water volume, and temperature [20,21]. Hay is usually soaked for 15–60 min, and extending this time not only reduces nutrient content but also significantly decreases feed palatability [18,20,22]. Even in healthy horses, leaching NSC from hay is advisable only when its level exceeds 10%. However, as hay is often purchased from various sources, analyzing its composition seems pointless, whereas preventive hay soaking may bring the expected benefits. It is not known, however, whether such treatments affect the emergence of undesirable feeding behaviors in horses, e.g., the unwillingness to consume this feed.

Technologies for combining hay with water are being introduced despite awareness of many adverse effects, such as accelerated putrefaction [23,24]. This is because such hay becomes an ideal substrate for faster and up to five times greater growth of bacteria and fungi within a relatively short time compared to dry hay. In order to prevent this negative effect on horses’ health, it is necessary to increase the labor involved in the frequent removal of unconsumed feed, the preparation of which is also time-consuming.

Soaked hay is a component of the diet for horses with metabolic problems [25,26]. An increasing proportion of the domestic horse population comprises obese horses with equine metabolic syndrome and insulin dysregulation, in which excessive consumption of NSCs can lead to recurrent laminitis [27]. Particularly vulnerable are horses and ponies, which are genetically adapted to environments with scarce food [28]. In animals suffering from polysaccharide storage myopathy (PSSM), it is also necessary to restrict NSCs [29,30]. Horses affected by a genetic condition known as hyperkalemic periodic paralysis (HYPP), i.e., Quarter Horses, American Paint Horses, Appaloosas, and Quarter Horse crossbreeds, must have their potassium (K) intake restricted [31,32].

This study assumed that moistening or soaking hay would not change the feeding behavior of Polish Konik horses, which are not selective feeders but are unaccustomed to such hay. It was also expected that moistening the hay would increase its palatability, leading to a significant reduction in the time required to consume the entire portion of this feed compared to dry or soaked hay. Based on this hypothesis, the aim of the study was to assess the time and frequency parameters characterizing the feeding behavior of the horses, as well as the mass of feed refusals left after the provision of dry, moistened, and soaked hay.

## 2. Materials and Methods

### 2.1. Approval of the Ethics Committee

Pursuant to the Act on the protection of animals used for scientific or educational purposes [Journal of Laws of 2015, item 266], research requires approval if it “causes pain, suffering, distress or lasting harm to the animal’s body, to an extent equivalent to or greater than a needle prick”. The observations described in this paper do not bear the above-mentioned characteristics. The study was conducted with the consent of the animals’ owners as part of routine desensitization to environmental stimuli.

### 2.2. Horses

The research material consisted of 12 adult Polish Konik horses, including six mares and six geldings, kept in a stud farm in the Roztocze National Park (RNP, Poland). The average age of the horses was 8.50 ± 1.84 years, and body mass was 327 ± 17.50 kg. During the experiment, the horses exhibited no symptoms of disease or behavioral abnormalities. No malocclusion or other oral anomalies, including missing teeth, were observed in the animals.

During the off-pasture period, the horses were fed three times a day (at 8.00 AM, 3.00 PM, and 9.00 PM) with 2 kg of dry meadow hay at each feeding. The hay was a mixture of selected grass varieties from RNP meadows, grown in semi-mineral soil with a pH of 6.5. The horses were, therefore, accustomed to this hay. Organoleptic assessment of the hay from the first cut, taken during the middle of the flowering stage, was positive. The hay was noted to have a pleasant aroma, a yellow-green color, low stem brittleness, and no soil contamination. The chemical composition of hay with a moisture content of 9.2% was as follows (g/kg DM): crude protein 76.0, NSC 130.0, crude fiber 350.0, ash 31.2, crude fat 11.5, Ca 3.48, and P 2.46. Furthermore, the horses had constant access to water and mineral blocks of NaCl supplemented with Ca and P.

During the off-pasture period, the horses were kept in open-air runs near the stables between 9.00 AM and 2.30 PM. For the remaining part of the day, the horses were kept in a stable divided into stalls. Wheat straw was used as bedding. During the study, the horses did not work.

### 2.3. The Course of the Experiment

The experiment was conducted during the last days of April (before grazing season) in three rounds (one round per day), each involving four horses kept in adjacent stalls. Each round lasted three consecutive days. During the study, all the horses were kept in a stable, in individual stalls, and bedded with wheat straw 30 min earlier. At that time, there was no hay in the stalls. The air temperature in the central part of the stall, at a height of 100 cm above the floor, was 13–15 °C, and the relative humidity ranged from 67 to 76%.

For three days prior to the beginning of the experiment, the horses were allowed to become accustomed to black, circular plastic containers with a diameter of 0.8 m and a height of 0.3 m, which were placed on the bedding at the spot where hay was normally provided. These containers were used to provide portions of hay during the experiment. One hour before feeding, the containers were scalded and dried in the feed room. Portions of hay were then prepared there and placed in plastic bags for weighing. The portion of dry hay was set at 1 kg as fed (measurement accuracy of 0.01 kg).

During the experiment, hay was provided every day at 8.00 AM as part of the daily feeding routine. This was done to minimize disturbances in feeding behavior.

On the first day, dry hay was provided (control test). On the second and third days, the weighed hay was placed, prior to its provision, in special round polypropylene nets. The hay was sprayed with running water using a garden sprinkler, with the water jet directed evenly over the entire surface of the net: for 1 min on the second day of the experiment (moist hay) and for 15 min on the third day (soaked hay). Groundwater at 5 °C was used. The hay was weighed again; the moist hay weighed 2.1 ± 0.14 kg, and the soaked hay weighed 3.3 ± 0.18 kg.

The hay prepared in this way was provided in containers placed on the bedding in a stall at the usual hay feeding spot, next to the drinker, to all four horses simultaneously. The experiment began when the observers left the stalls, i.e., approximately 5 s after the hay was provided. After a further one hour, the containers were removed (a maximum time). The moment when the observers returned to the stalls signaled the end of the experiment. The horses that were not examined on the particular day were provided with a standard portion of hay. The horses being examined, however, were provided with additional hay after the observation period ended.

### 2.4. Research Methods

During the experiment, time and frequency measurements of feeding behavior were collected by the authors (a total of four people across three three-day rounds). These individuals were located in the middle of the stable corridor without making physical or vocal contact with the horses. No case of a horse showing interest in the observers was noted during the study. The time parameters (s) were measured using a digital stopwatch from the start of the study until the maximum time was reached. These included (1) hay intake time: the total time spent by each animal putting its muzzle into the container for at least 3 s, picking up hay scattered by the horse, and chewing the hay; (2) duration of pauses in hay intake (this only applies to situations where the horse was standing by the hay container): the total time of each completion of hay intake until the horse puts its muzzle back into the container for at least 2 s or begins to pick up hay from the bedding; (3) straw intake time: the total time of each straw intake; and (4) drinking time: the total time of each instance of water drinking, measured from the moment the horse places its muzzle in the drinker for at least 2 s until the muzzle is removed from the drinker.

The frequency characteristics included (1) frequency of pauses in hay intake: the number of events involving the completion of hay chewing until the moment the horse places its muzzle back into the container for at least 3 s or the horse begins to pick up hay from the bedding (this only applies to situations where the horse was at the container and was not eating straw); (2) drinking frequency: the number of events involving the horse placing its muzzle in the drinker for at least 2 s.

The feed refusals left in and around the container were dried and then packed into a plastic bag. The mass of feed refusals was then determined (with an accuracy of 0.01 kg).

### 2.5. Statistical Methods

Statistical analysis of the data was performed using Statistica for Windows 13.0 (TIBCO Software Inc., Palo Alto, CA, USA). The main analysis compared variables according to hay type (dry, moist, soaked). The horse’s sex was also considered. The normality of the data distribution was assessed using the Shapiro–Wilk test. After checking for homogeneity of variance (Levene’s test), the variables for which normal distribution was confirmed (‘hay intake time’ and ‘duration of breaks in hay intake’) were analyzed using one-way analysis of variance. The significance of differences between mean values was determined using Tukey’s test (RIR). For the remaining variables, due to non-conformity with the normal distribution, Friedman’s rank test and the Wilcoxon signed-rank test were employed. For comparisons between sexes, the *t*-test for independent samples was used when the data were normally distributed, and the Mann–Whitney U test was used when the data were not normally distributed. The results are presented as means ± SD, with min and max. The differences between the groups were considered significant at *p* ≤ 0.05. To determine the relationships between the variables under study, Spearman’s correlations were used for each type of hay (*p* ≤ 0.05).

Furthermore, for each horse, scores ranging from 1 to 6 were assigned for a trait defined as ‘hay palatability’. The following scoring algorithm was established: 1 point: no palatability (hay intake time is shorter than or equal to the mean, and the mass of feed refusals is greater than the mean); 2 points: very low palatability (hay intake time is longer than the mean, and the mass of feed refusals is greater than the mean); 3 points: low palatability (hay intake time is longer than the mean, and the mass of feed refusals is less than or equal to the mean); 4 points: moderate palatability (hay intake time is shorter than or equal to the mean, and the mass of feed refusals is less than or equal to the mean); 5 points: high palatability (hay intake time is longer than the mean, and there are no feed refusals); 6 points: very high palatability (hay intake time is shorter than or equal to the mean, and there are no feed refusals). This trait was determined separately for each type of hay taken by the mares and the geldings.

For the horses as a whole, their numbers and the percentage proportion were indicated on the basis of the points assigned for ‘hay palatability’. The decision to present the results for all horses collectively was based on the lack of significant differences between the sex groups’ means and the small number of horses, which is considered a weakness of the manuscript.

## 3. Results

**Figure 1:**
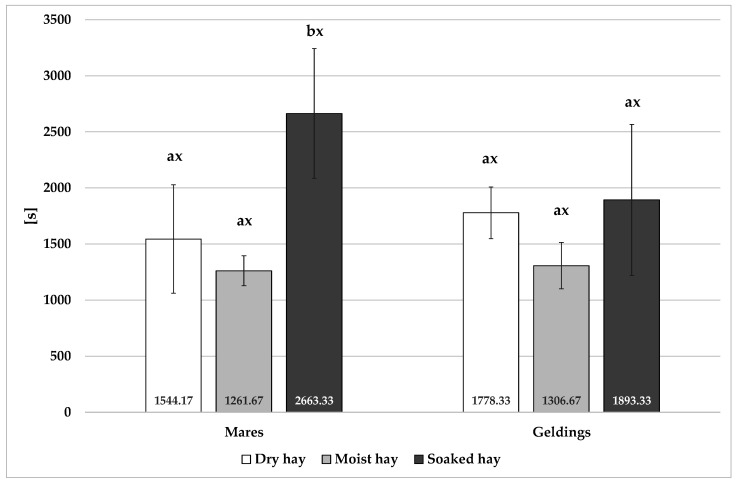
Time (s) of hay intake by the horses. Means denoted by different letters differ significantly at *p* ≤ 0.05 (a, b: a comparison between different types of hay; x: a comparison between sexes).

The time of hay intake by the mares was significantly longer than that for the geldings in the case of soaked hay (Figure 1). In the gelding group, no significant differences were noted. No sex-related differences were observed.

**Figure 2:**
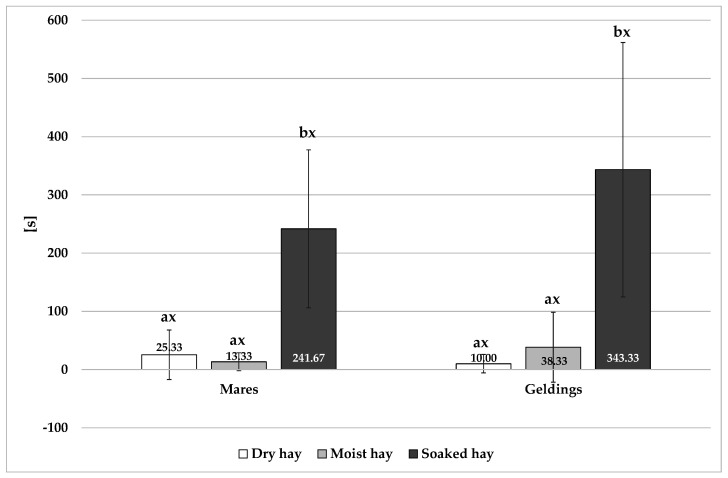
Dry matter (g) of hay refusals left by the horses. Means denoted by different letters differ significantly at *p* ≤ 0.05 (a, b: a comparison between different types of hay; x: a comparison between sexes).

In both the mare and gelding groups, the mass of soaked hay refusals was significantly greater than the mass of dry and moist hay refusals (Figure 2), and no sex-related differences were observed.

**Figure 3:**
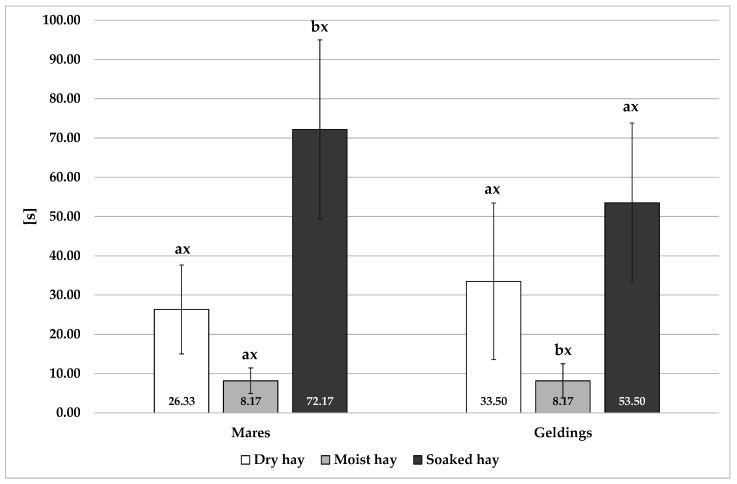
Duration (s) of pauses in hay intake by the horses. Means denoted by different letters differ significantly at *p* ≤ 0.05 (a, b: a comparison between different types of hay; x: a comparison between sexes).

Significant differences were observed in the ‘duration of pauses in hay intake’ trait for both the mares and the geldings (Figure 3). During the intake of soaked hay by the mares, this trait took on a value significantly greater than that during the intake of dry or moist hay. In the gelding group, this time was significantly shorter than in the other groups during the intake of moist hay. No sex-related differences were noted.

Significant differences in the frequency of pauses in hay intake only occurred in the mares. During the intake of dry hay, the mean was significantly lower than that noted during the intake of soaked hay. However, during the provision of moist hay, this trait took on intermediate values. No sex-related differences were noted. Differences in the time of straw intake occurred in both sex groups, with the mean value being significantly greater during soaked-hay intake by the horses. Furthermore, for the intake of moist hay by the mares, not a single instance of straw intake was noted. No sex-related differences were noted.

**Figure 4:**
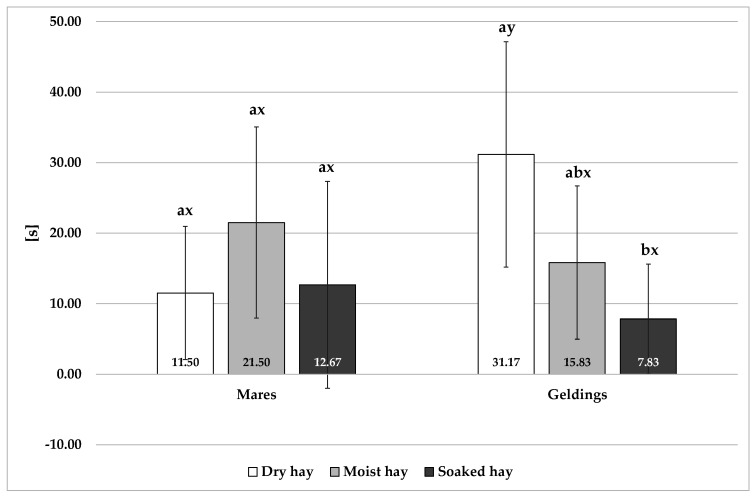
Time (s) of water drinking by the horses. Means denoted by different letters differ significantly at *p* ≤ 0.05 (a, b: a comparison between different types of hay; x, y: a comparison between sexes).

Significant differences in drinking time during the provision of various types of hay to horses were only observed for the geldings (Figure 4). This trait, during the provision of dry hay, was significantly greater than that noted during the provision of soaked hay. When moist hay was provided, this trait took on an intermediate value. Sex-related differences were also noted, but only during the provision of dry hay. At that time, the geldings drank water for significantly longer than the mares.

The frequency of drinking during the provision of dry, moist, and soaked hay did not differ significantly in either the mare or the gelding groups, and no sex-related differences were observed.

A significant correlation between the traits under analysis during the provision of dry hay to the horses was noted in 1 out of 21 possible cases, accounting for 4.76%. The time of hay intake was negatively correlated with the mass of feed refusals.

Significant correlations between the traits under analysis during the provision of moist hay to the horses were noted in 3 out of 21 possible cases, accounting for 14.28%. The duration of pauses in feed intake was positively correlated with the time and frequency of drinking. Furthermore, drinking frequency and drinking time were also positively correlated.

Significant correlations between the traits under analysis during the provision of soaked hay to the horses were noted in 4 out of 21 possible cases, accounting for 19.04%. These concerned the relationship between the duration of breaks in hay intake, the duration of straw intake and the duration of water drinking, and the frequency of water drinking, as well as the duration of straw intake and the duration of water drinking.

**Figure 5:**
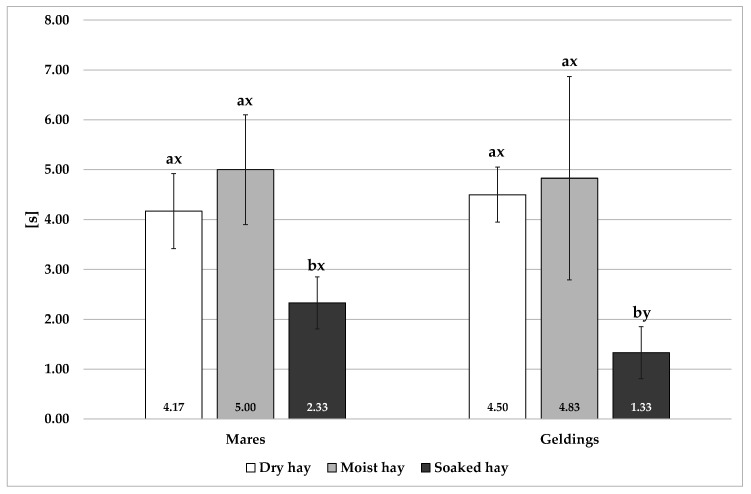
The average score assigned for hay palatability. Means denoted by different letters differ significantly at *p* ≤ 0.05 (a, b: a comparison between different types of hay; x, y: a comparison between sexes).

The average score for ‘palatability’ of hay was significantly lower for both the mares and the geldings during the intake of soaked hay, compared with the scores for ‘palatability’ of dry and moist hay (Figure 5). Furthermore, the score for this trait during the intake of soaked hay by the mares was significantly higher than that for the geldings.

During the intake of dry hay, the majority, i.e., almost 90% of the horses, found it to be moderately or highly palatable (Table 1). For 8% of the remaining horses, palatability was low. After providing moist hay, the results were completely different. The majority, i.e., 58% of the horses, found the ‘palatability’ to be very high. In the group of 33% of the horses, however, a moderate ‘palatability’ was noted. There was also only one case of a horse for which moist hay was not palatable. For soaked hay, all of the horses displayed varying levels of ‘palatability’: for 33% of the horses, it was not palatable at all; 50% of them showed very low ‘palatability’; and for 17% of the horses, soaked hay’s ‘palatability’ was low.

## 4. Discussion

When developing the methodology for the experiment, we were aware that the time of feed intake, without comparing it with the mass of feed refusals, would only indicate possible changes in the horses’ feeding behavior after providing them with hay mixed with water compared with dry hay. Other authors investigating the use of various types of feed for horses considered the time of intake as an indicator of palatability, but when feed refusals occurred, the maximum time was assigned [33]. We, however, deliberately did not introduce such a rule, as it would have distorted the actual behavioral picture we aimed to examine. We observed such behavioral changes in the mares after the provision of soaked hay. It appears that providing them with specifically soaked hay resulted in a significant increase in the time spent consuming it, compared to dry or moist hay. Apparently, soaked hay was less palatable to the mares. No such differences were observed in the geldings, but to obtain unambiguous information, it was necessary to compare the results with the mass of feed refusals.

When comparing the time of feed intake with the mass of feed refusals, the results became clearer: for both mares and geldings, the provision of soaked hay was associated with leaving a significantly greater mass of feed refusals than when dry or moist hay was provided. Earing et al. [34] noted that soaking hay changes its nutrients, including losing non-structural carbohydrates (NSCs), which improve hay taste. We suggest, however, that the mares were, after all, less particular about feed than the geldings. It is possible that they picked out the tastier plant species from the generally unpalatable hay. At the same time, the geldings simply ceased consumption. Janczarek et al. [35] pointed out that horses of different sexes exhibit different levels of feed selectivity. The authors noted that mares were less particular about their feed than geldings and stallions.

At this stage of the discussion, we emphasize that soaked hay was not palatable to the horses. It would be interesting to investigate the palatability of soaked hay among horses accustomed to it and for horses of different breeds. The Polish Konik horse breed belongs to the group of primitive horse breeds, which have, among other things, modest nutritional requirements and are practically never particular about feeding [36]. Therefore, it can be assumed that similar results from studies on thoroughbred horses would be significantly more varied. As indicated by the results of a study conducted by Oberlin et al. [37], although the soaking of hay had no effect on the overall intake of dry matter, the time taken to consume it increased. Furthermore, horses not previously accustomed to steamed hay tended to consume larger quantities of it, compared to the horses that were accustomed to it, while the horses familiar with soaked hay tended to consume larger quantities of it, compared to the horses unfamiliar with it. These authors concluded that when modifying the type of hay or the method of its introduction into a horse’s diet, a period of adaptation is necessary to ensure that the horse consumes this feed in quantities that meet its nutritional requirements.

Turning now to the ‘palatability’ aspect, we believe that the descriptive scoring system yielded clearer results. It appears that dry hay was moderately or highly palatable to the horses. As before, we considered these results as controls and continued the experiment. The provision of moist hay to the horses considerably changed the distribution of scores. For more than half of the horses under study, we recorded the highest score, i.e., a very high palatability. This was, therefore, a novelty indicating the emergence of a dietary preference specifically towards moist hay. The intake of this hay by the geldings was also associated with the shortest and least frequent breaks. For the remaining horses, the palatability remained at a moderate level, i.e., similar to that for the palatability of dry hay.

It was also found that the only horse with a result of ‘low palatability’ of dry hay subsequently received a ‘no palatability’ score for both moist and soaked hay, suggesting that it may have been affected by a dietary issue (e.g., appetite disorders, silent colic) despite the absence of symptoms or there were other issues we were unaware of. After providing soaked hay, different results were obtained, indicating a significant decrease in the perceived palatability of this feed, with the vast majority of the horses rating it as having no palatability or very low palatability. Owens et al. [26] share this view regarding the palatability of soaked hay. Only two individuals received scores indicating moderate palatability. Furthermore, when the mares were fed soaked hay, the duration and frequency of breaks were significantly greater than when they were fed dry or moist hay. Therefore, these results indicate that the Polish Konik horses, not accustomed to the particular feed, are selective eaters, which does not support the results obtained by Chodkiewicz [36].

In studies conducted by Vasco et al. [38], Rodiek and Jones [39], and Cuddeford et al. [40], the feeding behavior of horses fed hay from different plants was compared, and it was found that alfalfa hay was the most palatable. Unfortunately, in the case of primitive Polish Konik horses, alfalfa in the form of green fodder or hay, as a highly nutritious feed, should not be used. It may contribute to the development of obesity, impaired renal and hepatic function, equine metabolic syndrome, and insulin dysregulation, ultimately leading to recurrent laminitis [25,26].

The effect of hay soaking was investigated by van den Berg et al. [41], Merkies and Bogart [42], and Danel and Merkies [43]. These authors emphasized that changes in the nutrient content of the feed, occurring at that time, could affect its flavor. Due to horses’ preference for sweet flavors, the loss of NSC through soaking and evaporation may reduce the palatability of hay. Also, according to Owens et al. [26], soaking hay reduced nutrient content, including NSC and potassium, which most likely led the horses to prefer dry or steamed hay over soaked hay.

Another confirmation of the unwillingness of Polish Konik horses, unaccustomed to soaked hay, to consume such hay was the time of straw intake. It appears that, overall, straw was consumed for a very short time, i.e., barely less than a minute, during our 60 min observations. Although they observed a significantly longer intake time when soaked hay was provided, they do not consider these results groundbreaking.

It was also noted that when soaked hay was provided, the geldings drank less water, which makes sense, as the hay contained extra water. Although there were no differences in this trait among the mares under study, and no sex-related differences were observed, we concluded that the results of the present study are generally consistent with those published by Oberlin et al. [37]. This is because these authors demonstrated that horses, regardless of sex, drank more water when fed dry hay compared to those fed soaked hay. No significant differences, however, were observed in drinking frequency.

To summarize this part of the discussion, it is also worth highlighting the marked individual differences between horses in many of the traits studied, as indicated by the high standard deviations and wide range of extreme values.

The traits under analysis correlated to a small extent, particularly when dry hay was provided to the horses. At this point, however, it is worth noting the relationship between the time of dry hay intake and the mass of feed refusals, which we found surprising, as it occurred only when dry hay was fed. Therefore, the absence of such relationships for the provision of moist or soaked hay was attributed to greater individual variation among the horses. However, what is puzzling is the correlation between the time of straw intake and the duration of activities unrelated to feeding behavior and the frequency of drinking. These results are difficult to interpret at this stage of the research.

## 5. Conclusions

The Polish Konik horses’ lack of familiarity with hay soaked in water most likely reduced their willingness to consume it. Food selectivity was primarily manifested by the observed feed refusals, which were generally not noted following the provision of dry or moist hay. The low palatability of soaked hay was more evident in the geldings, which consumed this feed for shorter periods and in smaller quantities than the mares. Furthermore, the palatability of moist hay appeared to be higher than or comparable to the palatability of dry hay. However, the reduced willingness to consume hay did not contribute to an increase in straw intake, as straw is not a substitute for roughage. It is also important to note that different results may be expected in some horses, as the values for the traits studied indicated considerable individual variation.

## Figures and Tables

**Table 1:** The number and percentage proportion of horses with different scores indicating ‘hay palatability’.

The Score for Hay Palatability	The Number of Horses	%
Dry hay		
1: no palatability	0	0
2: very low palatability	0	0
3: low palatability	1	8.33
4: moderate palatability	6	50.00
5: high palatability	5	41.66
6: very high palatability	0	0
Moist hay		
1: no palatability	1	8.33
2: very low palatability	0	0
3: low palatability	0	0
4: moderate palatability	4	33.33
5: high palatability	0	0
6: very high palatability	7	58.33
Soaked hay		
1: no palatability	4	33.33
2: very low palatability	6	50.00
3: low palatability	2	16.66
4: moderate palatability	0	0
5: high palatability	0	0
6: very high palatability	0	0

## Data Availability

The raw data supporting the conclusions of this article will be made available by the authors upon request.
